# Automated Lung Nodule Detection and Classification Using Deep Learning Combined with Multiple Strategies

**DOI:** 10.3390/s19173722

**Published:** 2019-08-28

**Authors:** Nasrullah Nasrullah, Jun Sang, Mohammad S. Alam, Muhammad Mateen, Bin Cai, Haibo Hu

**Affiliations:** 1 Key Laboratory of Dependable Service Computing in Cyber Physical Society of Ministry of Education, Chongqing University, Chongqing 400044, China; 2 School of Big Data & Software Engineering, Chongqing University, Chongqing 401331, China; 3 Department of Software Engineering, Foundation University Islamabad, Islamabad 44000, Pakistan; 4 Frank H. Dotterweich College of Engineering, Texas A&M University—Kingsville, Kingsville, TX 78363-8202, USA

**Keywords:** clinical biomarkers, deep convolutional neural networks, internet of things, pulmonary nodules, wireless body area networks

## Abstract

Lung cancer is one of the major causes of cancer-related deaths due to its aggressive nature and delayed detections at advanced stages. Early detection of lung cancer is very important for the survival of an individual, and is a significant challenging problem. Generally, chest radiographs (X-ray) and computed tomography (CT) scans are used initially for the diagnosis of the malignant nodules; however, the possible existence of benign nodules leads to erroneous decisions. At early stages, the benign and the malignant nodules show very close resemblance to each other. In this paper, a novel deep learning-based model with multiple strategies is proposed for the precise diagnosis of the malignant nodules. Due to the recent achievements of deep convolutional neural networks (CNN) in image analysis, we have used two deep three-dimensional (3D) customized mixed link network (CMixNet) architectures for lung nodule detection and classification, respectively. Nodule detections were performed through faster R-CNN on efficiently-learned features from CMixNet and U-Net like encoder–decoder architecture. Classification of the nodules was performed through a gradient boosting machine (GBM) on the learned features from the designed 3D CMixNet structure. To reduce false positives and misdiagnosis results due to different types of errors, the final decision was performed in connection with physiological symptoms and clinical biomarkers. With the advent of the internet of things (IoT) and electro-medical technology, wireless body area networks (WBANs) provide continuous monitoring of patients, which helps in diagnosis of chronic diseases—especially metastatic cancers. The deep learning model for nodules’ detection and classification, combined with clinical factors, helps in the reduction of misdiagnosis and false positive (FP) results in early-stage lung cancer diagnosis. The proposed system was evaluated on LIDC-IDRI datasets in the form of sensitivity (94%) and specificity (91%), and better results were obatined compared to the existing methods.

## 1. Introduction

Lung cancer is one of the deadliest cancers worldwide. However, the early detection of lung cancer significantly improves survival rate. Cancerous (malignant) and noncancerous (benign) pulmonary nodules are the small growths of cells inside the lung. Detection of malignant lung nodules at an early stage is necessary for the crucial prognosis [1]. Early-stage cancerous lung nodules are very much similar to noncancerous nodules and need a differential diagnosis on the basis of slight morphological changes, locations, and clinical biomarkers [2]. The challenging task is to measure the probability of malignancy for the early cancerous lung nodules [3]. Various diagnostic procedures are used by physicians, in connection, for the early diagnosis of malignant lung nodules, such as clinical settings, computed tomography (CT) scan analysis (morphological assessment), positron emission tomography (PET) (metabolic assessments), and needle prick biopsy analysis [4]. However, mostly invasive methods such as biopsies or surgeries are used by healthcare practitioners to differentiate between benign and malignant lung nodules. For such a fragile and sensitive organ, invasive methods involve lots of risks and increase patients’ anxieties.

The most suitable method used for the investigation of lung diseases is computed tomography (CT) imaging [5]. However, CT scan investigation has a high rate of false positive findings, with carcinogenic effects of radiations. Low-dose CT uses considerably lower power radiation contact than standard-dose CT. The results show that there is no significant difference in detection sensitivities between low-dose and standard-dose CT images. However, cancer-related deaths were significantly reduced in the selected population that were exposed to low-dose CT scans as compared to chest radiographs, which is depicted in the National Lung Screening Trial (NLST) database [6]. The detection sensitivity of lung nodules improves with sophisticated anatomical details (thinner slices) and better image registration techniques. However, this increases the datasets to very large extent. Depending upon the slice thickness, up to 500 sections/slices are produced in one scan [7]. An experienced radiologist takes approximately 2–3.5 min to observe a single slice [8]. The workload of a radiologist increases significantly to screen a CT scan for the possible existence of a nodule. In addition to section thickness of the CT slices, detection sensitivity also depends on nodule features such as size, location, shape, adjacent structures, edges, and density. 

Results show that only 68% of the time lung cancer nodules are correctly diagnosed when only one radiologist examines the scan, and are accurately detected up to 82% of the time with two radiologists. The detection of cancerous lung nodules at an early stage is a very difficult, tedious, and time-consuming task for radiologists. Screening a lot of scans with care requires plenty of time by the radiologist, meanwhile it is very much error-prone in the detection of small nodules [9].

In this situation, a tool is needed to assist radiologists by reducing reading time and detection of missed nodules, and allowing for better localization. Computer-aided detection (CAD) systems were initially designed to reduce the workload of radiologists and increase nodule detection rate. However, the latest generation of CAD systems also helps in the screening process by differentiating between benign and malignant nodules [10]. With the recent advances in deep neural networks, especially in image analysis, CAD systems are consistently outperforming expert radiologists in both nodule detection and localization tasks. However, results from various researchers show a wide range of detection from 38–100%, with a FP rate from 1–8.2 per scan by the CAD systems [11]. The classification between benign and malignant nodules is still a challenging problem due to very close resemblance at early stages.

**Figure 1:**
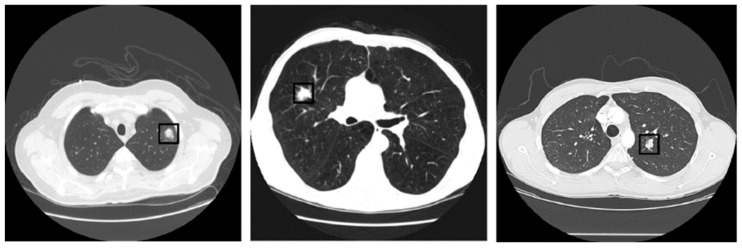
Categories of lung nodules in a CT scan; benign, primary malignant, and metastatic malignant (from left to right).

Benign and malignant nodules have considerable feature overlaps, but still have to be differentiated on the basis of morphology and location at early stages. Benign nodules are usually located at the peripheral, with smooth surfaces and triangular shapes filled with fat and calcium, while malignant nodules often show speculations with edges, lobulated, vascular convergence, cystic air spaces, pleural indentations, bubble-like lucencies, and sub-solid morphology. Malignancy is also related with size and growth of the nodules [12]. The three different categories (benign, primary malignant, and metastatic malignant) of lung nodules are shown in Figure 1.

Various types of errors can occur during the screening process. These are scanning errors, which lead to failure in capturing the lesion area, recognition error related to the failure in identifying the lesion, and decision-making error, which happens due to incorrect understanding of the benign and malignant lesions and the normal structures. In the majority of patients, these errors lead to delayed and wrong diagnoses, which are the main cause of mortality. Almost 4% of radiological reports comprehend diagnostic errors on a daily basis, and around 30% of abnormal radiological findings are missed. To counteract these errors, multiple strategies (deep learning-based CT scan analysis with clinical and physiological findings) have been used in connection for the detection and classification of early-stage lung nodules [13]. 

In our prior study, which is mentioned at the end of Section 2, we used a deep learning-based model for the diagnosis of early-stage lung cancer by CT scan analysis. However, due to the close resemblance of benign and maliganat nodules at early stages and various types of scanning errors, a large number of false positive results are reported in CT scan analysis techniques. To reduce false positive results, better deep learning-based models for nodule detection and classification have been developed in connection with clinical and physiological settings. A number of modifications have been done in our previous deep learning models to learn the nodules’ features in better way. Classification results of deep learning model were evaluated, in connection with clinical and physiological findings, to reduce the false positive results. The MixNet architecture used in previous work was modified to learn nodules’ features at a finer level.

The most popular modern CNN architectures are residual network (ResNet), densely connected network (DenseNet), dual path network (DPN), and mixed link network (MixNet). ResNet  is the winner of the ImageNet Large Scale Visual Recognition Competition (ILSVRC) 2015 (Image Classification, Localization, Detection). ResNet introduces skip connection (or shortcut connection) to fit the input from the previous layer to the next layer, without any modification of the input. Skip connection enables a deeper network, and finally ResNet became the winner of ILSVRC 2015 in image classification, detection, and localization, as well as the winner of MS COCO 2015 detection and segmentation. DenseNet paper got the best paper award of computer vision pattern recognition (CVPR) in 2017. DPN (Dual Path Network) is the combination ResNet and DenseNet. Mixed link networks have also shown that both dense connections and residual connections belong to a common topology. These methods utilize these interpretations to design hybrid networks that incorporate the core idea of DenseNet with that of ResNet. These works demonstrate that inclusion of addition and concatenation-based connections improves classification accuracy, and is more effective than going deeper or wider.

With the rapid growth of the internet of things (IoT) and medical sensor devices, patients can be examined distantly and continuously by physicians through wireless body area networks (WBANs). WBANs are one of the key applications of IoT that provides tele-monitoring of patient health ubiquitously. The purpose of an IoT-based healthcare system is to connect physicians, patients, and nurses via smart devices. In WBANs, various implantable, wearable, and invadable sensing devices are placed on the patient’s body for continuous remote monitoring of the vital parameters. This is known as medical IoT, which provides the advantages of recording and analyzing patient data for diagnosis. These technological advances have upgraded pulmonary cancer detection and classification, using CT scan images, with the help of numerous computer-assisted detection systems (CADe) [14]. With the innovation of IoT, medical data is accessible via distant sensors over the internet. These technologies have enabled the health sector to acquaint with new methods for the diagnosis and detection of lung cancer.

In this paper, automated lung nodule detection and classification using deep learning with multiple strategies is proposed. The proposed system works on three-dimensional (3D) lung CT scans, along with physiological symptoms and the clinical biomarkers, to reduce false positive results and ultimately prevent invasive methods. Two deep 3D modern convolutional networks were designed for nodule detection and classification, respectively. Faster R-CNN with CMixNet and U-Net-like encoder–decoder was designed for nodule detection [15]. For the classification of the nodules, the gradient boosting machine (GBM) [16] with 3D CMixNet was used. Our designed framework for nodule detection and classification on the publically available data set LIDC-IDRI outperforms the other state of the art deep learning methods. Supreme false positive reduction was achieved through combining multiple strategies on the suspicious results of the deep learning model.

The rest of the article is prepared as follows. In Section 2, we discuss some related works. Motivations regarding our study are highlighted in Section 3. The proposed work is described in Section 4. In Section 5, experimental results are described. Finally, the conclusions presented in Section 6.

## 2. Related Works

Although the first computer-aided detection (CAD) system for lung nodule detection was designed in the late 1980s, these attempts were not appealing due to inadequate computational resources for advanced image analysis techniques at that time. After the invention of the graphical processing unit (GPU) and convolutional neural networks (CNN), the performance of computer-based image analysis and decision support system got a high boost. A lot of deep learning-based medical image analysis models have been proposed by researchers, and a few of the most relevant lung nodule detection and classification methods are mentioned here.

Setio et al. proposed a 3D fully convolutional neural network for FP reduction in lung nodule classification [17]. A 3D network was used to analyze the 3D nature of the CT scans to reduce wrong diagnosis, and weighted sampling was used to improve results.

Ding and Liao et al. used 3D Faster R-CNN for nodule detection to reduce false positive (FP) results of lung cancer diagnosis [18]. Faster R-CNN shows very good results for object detection. It was used with very deep modern CNN architecture, the dual path network (DPN), to learn the features of the nodules for classification [19].

Jiang Hongyang et al. designed group-based pulmonary nodule detection using multi patches scheme with Frangi filter to boost the performance [20]. Images from the two groups were combined and a four channel 3D CNN was proposed to learn the features marked by the radiologist. Their CAD system’s results show sensitivity of 80.06% with 4.7 FP for each scan, and sensitivity of 94% with an FP rate of 15.1.

Zhu Wentao et al. proposed automated lung nodule detection and classification models using 3D DPN with 3D faster R-CNN and gradient boosting machine (GBM) by learning spatial features of lung nodules [18]. After preprocessing of the whole chest CT scan, the lung volume was segmented. The segmented lung volume was analyzed by 3D faster R-CNN with DPN and U-Net-like encoder–decoder architecture for nodule detection. After detecting the nodules, suspected nodule regions were cropped to learn the finer level features of the nodules to classify with DPN and gradient boosting machine. The model shows a detection accuracy of 87.5% with an error rate of 12.5%. 

Masood et al. proposed deep fully convolutional neural network (DFCNet) for the detection and classification of pulmonary lung nodules in a CT image [14]. Initially the nodule was classified as either benign or malignant; after that, the malignant nodule was further classified into four sub-classes on the basis of the CT image and metastasis information obtained from the medical IoT network. 

Gu Yu et al. proposed 3D deep CNN with multiscale prediction strategies for the detection of lung nodules from segmented images [13]. The 3D CNN performs much better with richer features than 2D CNN. In addition to 3D CNN, a multiscale lung nodule prediction strategy was applied for the small nodules with cube clustering techniques. 

Zhao J et al. proposed a new method for lung segmentation and nodule detection by combining the features from CT and PET images [4]. They used a dynamic threshold-based segmentation method for lung parenchyma from CT scans and identified doubtful areas through PET scans. After that, they performed watershed-based segmentation techniques to find the suspected areas of nodules in the CT images. Later, a support vector machine was used to classify the nodules in the CT images through textual features and, lastly, PET images were used to validate the method. 

Dr. Silvestri and his research team have proposed proteomic classifiers, along with nodule features, to differentiate between small size benign and malignant lung nodules [3]. They have achieved very good results on 8–30 mm nodule sizes with a reduction of 40% in biopsies on benign nodules.

In [11], the authors proposed 3D-CNN for the classification of the volumetric benign and malignant lung nodules to reduce the false positive results in an automated lung nodule detection setup in the CT scans. 

After the popularity of convolutional neural networks (CNNs) in image analysis, different types of connectivity patterns were proposed by researchers to increase the performance of deep CNNs. Up until now, in the deep CNNs, dense topology structures ResNet, DenseNet [21], and DPNs performance is superior as compared to other ones, but there is still room for connection improvements in these topologies [22]. The MixNet architecture has improved connection structures with better features of extraction and reduced parameter redundancy [23]. 

In our previous work [24], we used MixNet for the first time for lung nodule detection and classification with GBM on publically available LUNA16 and LIDC-IDRI datasets, and achieved very good results of detection (94%) and specificity (90%). In these datasets, only the nodules of sizes greater than 3 mm were annotated by the three to four expert radiologists. However, in the case of an individual radiologist’s examination of a CT scan, nodules of sizes less than 6 mm are usually missed. CT scan analysis techniques are facing a lot of false positive results in the early stage of lung cancers diagnosis. Therefore, a multi-strategy-based approach is needed for early-stage lung cancer detection. 

## 3. Motivations

A lot of CT scan analysis techniques exist for lung nodule detection and classification, which have sensitivity of up to 94% but with low specificity and high FP rate in the classification of nodules. On the other hand, lung cancer screening studies show that blood tests have higher specificity and lower sensitivity as compared to imaging tests [25]. Unfortunately, currently, there is no single biomarker which is 100% sensitive and specific for lung cancer diagnosis. There are several types of biomarkers for cancer diagnosis, which are divided into genetic (mutations, changes in DNA and RNA expression), proteomic (changes in plasma proteins level and expression), and the presence of tumor, immune, and endothelial cells in the blood. 

Dr. Silvestri and his research team have proposed proteomic classifiers to differentiate between small size benign and malignant nodules [3]. They have designed a blood test by performing mass spectroscopy of the plasma proteins. Two proteins, named LG3BP and C163A, were identified as biomarkers for cancerous lung nodule identification. The level of these proteins in plasma, along with five risk factors (age, nodule size, smoking status, edge characteristics, and location), were used as classifiers and obtained very good results. They have achieved very good results on 8–30 mm nodule sizes and reduced biopsies on benign nodules by 40%. Table 1 shows the effect of each factor on the correct detection and diagnosis of lung nodules. The nodules’ features are better learned through computer-based deep learning methods as compared to radiologists. This motivated us to combine the results of an efficient deep learning model with biomarkers to reduce the false positive results of CT techniques, while the combination of multiple biomarkers provides high detection and diagnosis capability. Although a number of computer-based CT scan analysis techniques have better detection performance than the radiologists, this still does not address nodules of sizes <3 mm. Therefore, the final decision regarding early-stage lung cancer diagnosis should be performed by using multiple strategies, because approximately 60–80% of lung cancers are diagnosed at advance stages. 

## 4. Proposed System

**Figure 2:**
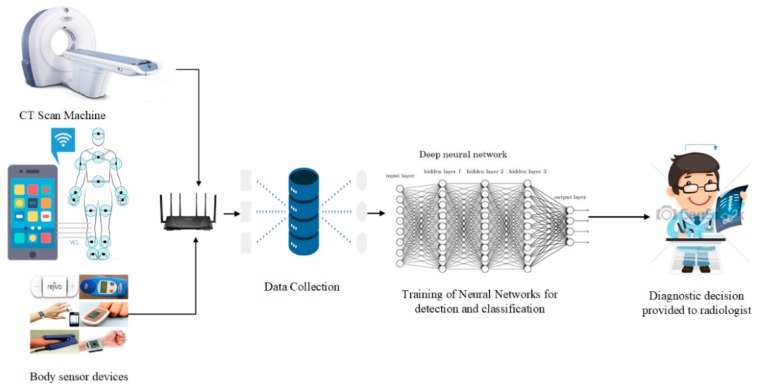
Overview structure of the proposed lung nodule detection and classification system.

**Figure 3:**
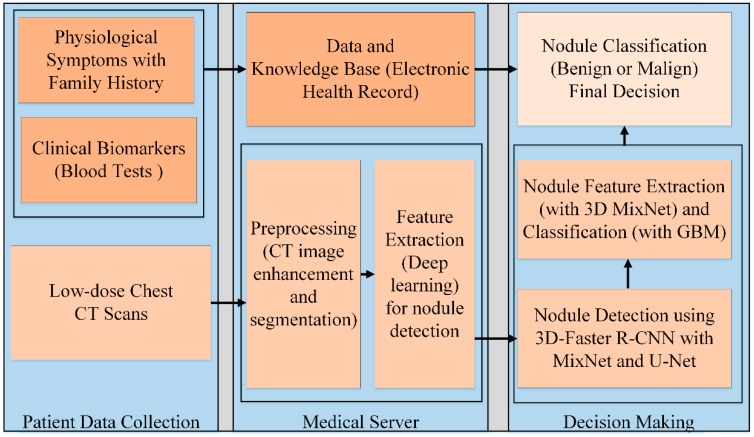
Block diagram of the proposed lung nodule detection and classification system.

The proposed automated lung nodule detection and classification system works on multiple strategies to decrease FP results. The system performs decisions based on physiological symptoms, CT scan analysis, and clinical biomarkers. UN-attempted physiological symptoms lead to lung cancer prognosis with family history about lung cancer. Deep learning-based CT scan analysis techniques outperform radiologists in the detection of lung nodules, especially of nodule sizes of <6 mm in diameter, but classification between benign and malignant nodules is a significant and challenging task due to considerable overlap of features. To reduce the negative predictive value, the proposed system performs decisions based on multiple strategies. Clinical biomarkers, especially plasma proteins and blood tests, are very useful for the classification of early-stage lung nodules. Figure 2 shows the overview structure of the proposed system, while Figure 3 demonstrates the block diagram of the proposed system. In Figure 3, the dark-orange blocks represent the physiological and clinical pathway through an IoT-based wireless body area network. The light-orange blocks represent the deep learning-based lung nodule detection and classification pathway. Finally the very light orange block shows the collective decision on the basis of physiological and CT scan examinations.

### 4.1. Monitoring Physiological Symptoms

Physiological symptoms of an individual can be continuously observed remotely using a wearable, invadable, and implantable IoT device. Symptoms’ appearances in lung cancer patients vary from stage-to-stage. The most common physiological symptoms that occur in the early stages of lung cancer are coughing, breathlessness, bronchitis, chest pain, wheezing, rust-colored phlegm, fatigue, body weight loss, difficulty in swallowing, pneumonia, and swelling of feet. However, most of the pulmonary cancer symptoms appear when it spreads out in the body. Such type of cancer is known as metastatic cancer, which shows symptoms such as backache, dizziness, seizures, numbness, yellowing of the skin, appearance of lumps in neck region, hypercalcemia, blood pressure, nausea, vomiting, constipation, fatigue, pain, confusion, anxiety, and disorders of the nervous system. The above symptoms are not restricted only to lung cancer; research results indicate that these may appear due to malignant lung nodules.

Chongqing (CHN.USA) Hygeia Cancer Hospital provided the clinical information of lung cancer patients. Physiological data was also gathered remotely and continuously through IoT-based sensor devices. Table 2 depicts the probabilities of various physiological symptoms at different stages. These are the common symptoms in the early stage of lung cancer but without any other indication of the symptoms, they lead to lung nodule examination. Some of the symptoms appear at advanced stages, which are used to determine the metastasis information of the lung cancer. In the case of metastasis lung cancer, continuous data observation through smart devices is very helpful to monitor the spread of the cancer. 

WBAN devices send body physiological information to the physician’s smartphone and PC in real-time scenario. This information can be processed remotely and better decisions can be made based on the electronic healthcare record through the intelligent system. The body sensor devices connect through smartphones to the IoT networks and to clouds where huge data can be processed for better decisions. In our proposed work, we used sensing devices and smartphone applications to gather the physiological information of the patient and send to the remote cloud for further processing and analysis.

Breathlessness patterns were acquired by observing the breathing index with Rejuven’s Rejiva [online]. For heart rate and other fitness information, the Runtastic smartphone application was used [online]. Blood-pressure (BP) was measured through wearable sensor devices and body temperature was obtained by a smartphone application. Due to the inaccessibility of these devices at the time of the research, we used these sensor devices at a healthcare center (in our case it was Chongqing (CHN.USA) Hygeia Cancer Hospital). For this research work, we used monitoring devices in the form of wearable or implantable devices and smartphone applications. Information regarding insomnia was obtained through recording sleeping pattern with the help of wearable devices. Body temperature was recorded through smartphone application “Finger print thermometer”. BP was measured with a wearable BP sensor, and body weight loss by health assistant application.

### 4.2. CT Scan Analysis Using Deep Learning

The proposed deep learning-based lung cancer diagnosis is the updated version of our previous work [24] and consists of two phases: Nodule detection and classification with maximum detection and accuracy objectives. We have made several modifications to our previously proposed model, such as group convolutions in the form of ResNeXt instead of ResNet, full pre-activation and better traning procedures to increase the accuracy of the deep learning method. 

#### 4.2.1. Nodule Detection with 3D Faster R-CNN and CMixNet

It is a significant and challenging problem to automatically detect and classify small lung nodules with an average size of 1cm^3^ in the segmented lung CT image of a size approximately 30 × 30 × 40 cm^3^. Therefore, sophisticated modern deep learning convolutional network models were used in connection for the detection and classification of small size lung nodules in the segmented CT scan images. The reconstructed volumetric CT image was divided into 96 × 96 × 96 patches due to the limitations of GPU in terms of memory and processing. Then, each patch was processed separately and the results of all the patches were combined for nodule detection.

**Figure 4:**
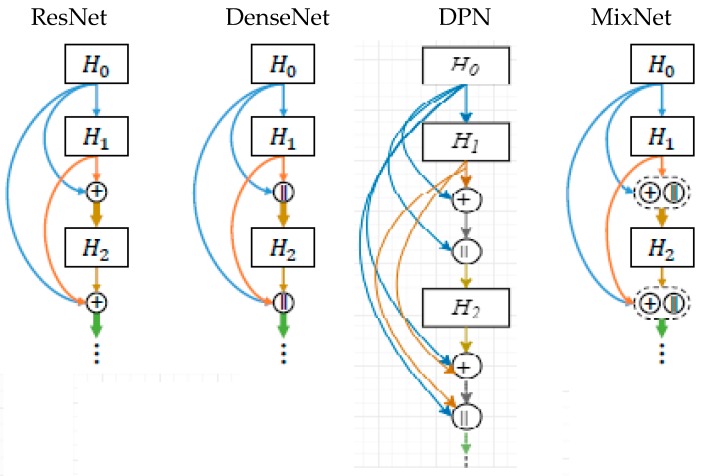
Modern CNN connection architecture.

The 3D Faster R-CNN-like architecture was used for lung nodule detection. Instead of simple CNN architecture, CMixNet with U-Net-like encoder–decoder architecture was used for learning nodules’ features. MixNet combines the advantages of well-known deep networks ResNet, DenseNet, and Dual path networks (DPNs) in an efficient way. MixNet is a special connection structure of ResNet and DenseNet that is slightly different from DPNs. The connection structures of these modern networks are shown in Figure 4. In ResNet, features of the previous layers element-wise are added up to make the features more expressive and to ease gradient flow for optimization simultaneously. However, too numerous additions over the same feature maps may encumber the flow of information in the network, which is the motivation to develop a “shifted additions”, by dislocating/shifting the additive positions in subsequent feature spaces along multiple layers. Therefore, in MixNet, ResNet is represented with an addition sign (“+”) that operates on the entire feature map. 

**Figure 5:**
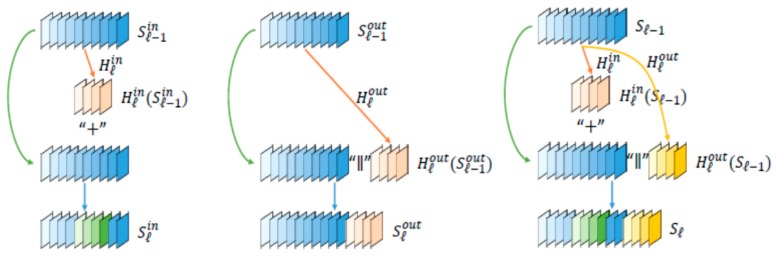
Inner link module (**left**), outer link module (**middle**), and mixed inner–outer module (**right**).

In DenseNet, raw features from the previous layers are directly concatenated to the new layer. Therefore, DenseNet is represented with a concatenation (“||”) operator. It improves the flow of information and gradually increases the feature dimensions along the depth. However, there may be the same type of features from different layers, which leads to a certain redundancy. This limitation is also the inspiration to introduce the “shifted additions” in MixNet. The mixed link connection structure is shown in Figure 5, as follows.

MixNet achieves superior performance in terms of parameter efficiency and results. The proposed MixNet works in the form of blocks, and each block has multiple L layers. Each layer has a nonlinear transformation function $H_{l}\left(.\right)$, where l represents the layer index. $X_{l}$ denotes the immediate output of the transformations $H_{l}\left(.\right)$, and $S_{l}$ is the output of the connection function C(.) whose inputs are the feature maps X from the previous layers, which are $\left(X_{0},X_{1},\ldots \ldots .,X_{l-1}\right)$. In dense topology, the connection function was used for connecting each layer with all the previous layers and can be formulated as follows:

(1) $$X_{l}=H_{l}\left(C\left(X_{0},X_{1},\ldots \ldots .,X_{l-1}\right)\right)$$

(2) $$X_{l}=H_{l}\left(X_{0}∥X_{1}∥\ldots \ldots ∥X_{l-1}\right)$$

While in ResNet, connection function was used for the addition of previous feature map to the current output. Flexible inner and outer link modules make the MixNet stronger. The inner link module (“+”) can be defined as follows:

(3) $$S_{l}^{in}=\sum_{i=0}^{l}X_{i}=S_{l-1}^{in}+X_{l}$$

(4) $$S_{l}^{in}=S_{l-1}^{in}+H_{l}^{in}\left(S_{l-1}^{in}\right)$$

where $H_{l}^{in}\left(.\right)$ refers to the function of producing feature maps for inner linking—element-wisely adding new features $H_{l}^{in}\left(S_{l-1}^{in}\right)$ inside the original ones $S_{l-1}^{in}$.

The outer link modules (“||”) are based on the concatenated connection. Similarly, we have $S_{l}^{out}$ as:

(5) $$S_{l}^{out}=X_{0}∥X_{1}∥\ldots \ldots ∥X_{l}=S_{l-1}^{out}∥X_{l}$$

(6) $$S_{l}^{out}=S_{l-1}^{out}∥H_{l}^{out}\left(S_{l-1}^{out}\right)$$

where, $H_{l}^{out}\left(.\right)$ refers to the function of producing feature maps for outer linking—appending new features $H_{l}^{out}\left(S_{l-1}^{out}\right)$ outside the original ones $S_{l-1}^{out}$.

The mixed link architecture can be formulated as (7), a flexible combination of (4) and (6), to get a blending feature output $S_{l}$.

(7) $$S_{l}=\left(S_{l-1}+H_{l}^{in}\left(S_{l-1}\right)\right)∥H_{l}^{out}\left(S_{l-1}\right)$$

where $H_{l}^{in}\left(.\right)$ and $H_{l}^{out}\left(.\right)$ represent the channel number of feature maps for inner $\left(k_{1}\right)$ and outer $\left(k_{2}\right)$ link modules, respectively. Combining these feature maps in MixNet can be defined as $\left(k_{1},k_{2},fixed/unfixed\right)$. The terms fixed and unfixed are used to control the position to merge the inner link features in the collective feature space. We used ResNeXt instead of ResNet in our CMixNet architecture, which is the major backbone network with thin layer DenseNet of growth rate k = 8. ResNeXt with cardinality C = 32 was used to learn features. The model compactness was achieved through bottleneck and transition layers. Bottleneck layers were implemented on the output of both inner and outer link modules with batch normalization (BN), ReLu non linearity, and convolution layers. The transition layer consists of transition down and transition up. Transition down comprises BN, Relu, 1 × 1 convolution, dropout, and non-overlapping max pooling, while transition up consists of 3 × 3 transposed convolution with stride 2 [21]. Such a compact DenseNet is also known as DenseNet-BC. The output of the feature maps of our CMixNet architecture at different stages are shown in Table 3.

The nodule detection task was performed through faster R-CNN that works at three stages, which are feature extraction, region proposal, and detection. Feature extractions were performed through state-of-the-art CMixNet, and region proposals were performed via U-Net-like encoder–decoder by pixel-wise labeling. The integration of CMixNet with U-Net makes it more robust for nodule feature extraction and generation. The encoder network with CMixNet also implemented batch normalization and dropout. Similarly, in the decoder network, up-sampling/deconvolution operations were performed with CMixNet architecture. Detection was performed through learned features and region proposal by inserting bounding boxes and finally classification. Three different sizes of anchor (5, 10, 20) were designed depending upon the distribution of nodules’ sizes in the annotated datasets. Each anchor had multiple factors in the loss functions. Classification loss $\left(L_{cls}\right)$ tells about the current box and whether it contains a nodule or not, while the regression loss $\left(L_{reg}\right)$ determines the diameter size d and coordinates (x,y,z) of the nodule. Anchor boxes were allotted labels based on intersection over union (IoU) values with ground truth boxes. If the overlap area of the detected and ground truth box was greater than 0.5, it was considered as positive with probability $\left(p^{*}=1\right)$, otherwise ground truth boxes with less probability were considered as negative with probability $\left(p^{*}=0\right)$. The loss function for an anchor i is defined as follows:

(8) $$L\left(p_{i},t_{i}\right)=\lambda L_{cls}\left(p_{i},p_{i}^{*}\right)+p_{i}^{*}L_{reg}\left(t_{i},t_{i}^{*}\right)$$

where $p_{i}$ denotes the predicted probability for an anchor i that contains a nodule, and $t_{i}$ is the relative coordinates predicted for current nodule position that is defined as:

(9) $$t_{i}=\left(\frac{x-x_{a}}{d_{a}},\frac{y-y_{a}}{d_{a}},\frac{z-z_{a}}{d_{a}},\log\left(\frac{d}{d_{a}}\right)\right)$$

where (x,y,z,d) represents the coordinates and diameter of the calculated nodule in the original space, and $\left(x_{a},y_{a},z_{a},d_{a}\right)$ denotes the coordinates and diameter of the anchor i. The ground truth nodule position is defined as:

(10) $$t_{i}^{*}=\left(\frac{x^{*}-x_{a}}{d_{a}},\frac{y^{*}-y_{a}}{d_{a}},\frac{z^{*}-z_{a}}{d_{a}},\log\left(\frac{d^{*}}{d_{a}}\right)\right)$$

where $\left(x^{*},y^{*},z^{*},d^{*}\right)$ represent the coordinates and diameter of the ground truth nodule. 

**Figure 6:**
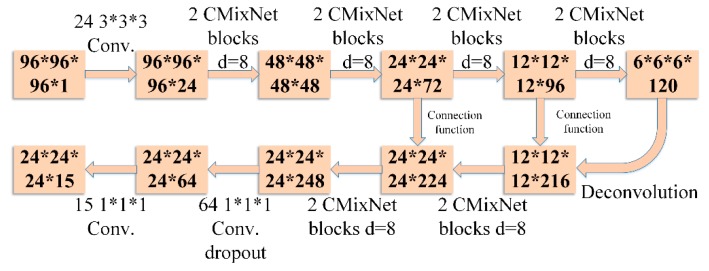
Feature extraction using CMixNet with U-Net-like encoder–decoder architecture for nodule detection. The numbers in the boxes represent the sizes of the feature maps in the sequence #slices, #rows, #cols, and #maps. The numbers outside the boxes are in sequence #filters, #slices, #rows, and #cols.

**Figure 7:**
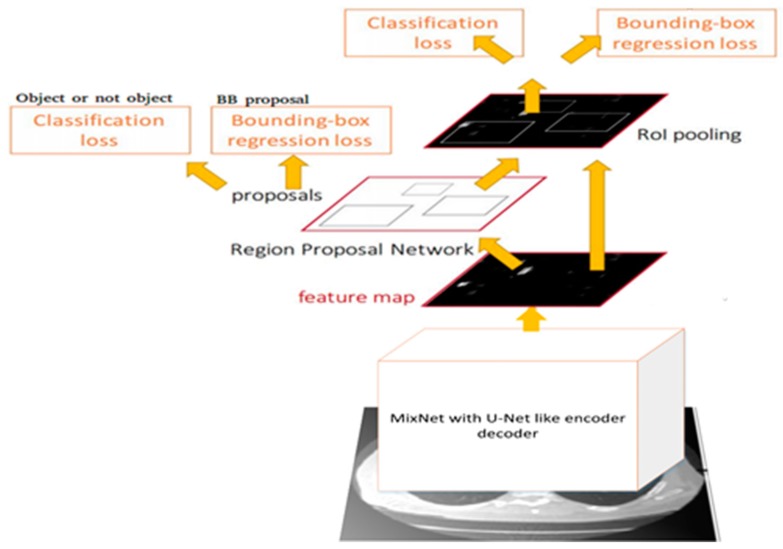
Nodule detection through Faster R-CNN.

The value of λ was set to 0.5. For $L_{cls}$, binary cross entropy loss function was used. For $L_{reg}$, regression loss function was used for smooth $l_{1}$. The feature extraction process for nodule detection is depicted in Figure 6. Using these features, nodule detection through faster R-CNN is shown in Figure 7.

The multi-task loss function comprises L1 and Focal Losses. The former is concerned with regression error, while the latter is used to evaluate classification error. 

#### 4.2.2. Nodule Classification Using GBM with CMixNet

**Figure 8:**
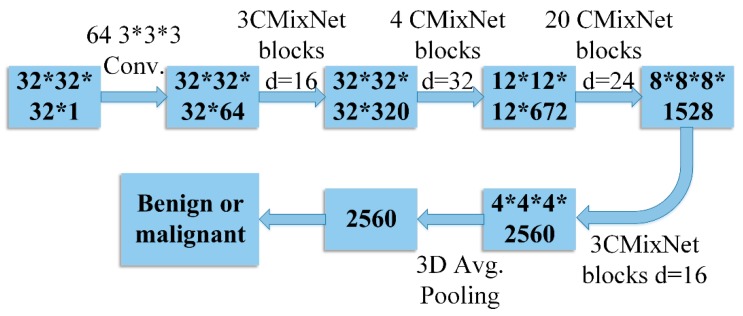
Nodule classification using CMixNet and GBM. The numbers in the boxes represent the sizes of the feature maps in the sequence #slices, #rows, #cols, and #maps. The numbers outside the boxes are in sequence #filters, #slices, #rows, and #cols.

Differentiating between early-stage malignant and benign lung nodules based on slight morphological changes is a challenging task and requires advanced deep learning techniques. Firstly, the lung CT images were cropped and centered for expected nodules with a dimension of 32 × 32 × 32. After that, modern convolutional neural networks were used for feature extraction. A modified 3D MixNet architecture (CMixNet) was used for feature extraction from the predicted lung nodules to classify the malignant and benign nodules in the CT images. Due to minute differences between benign and malignant lung nodules, adequate details are required to classify the nodules. Therefore, 30 3D CMixNet blocks were used to learn higher-level features. In the classification phase, after two convolution layers, average pooling was performed and logistic regression was used to differentiate benign and malignant nodules. GBM was also used on detailed features for classification. After constructing features from deep networks, GBM was applied, which obtained 87.21% accuracy in classification. The nodule classification process is shown in Figure 8. 

### 4.3. Major Clinical Biomarkers

Some clinical biomarkers are very helpful for the early diagnosis of lung cancer. Some of the blood tests show indications of malignancy, such as complete blood count, serum calcium level, antigens, and plasma proteins—especially LG3BP and L163A. The measurement of biomarkers is very helpful for the screening of cancer patients. Different types of biomarkers are used in connection with other risk factors for the possible evaluation of pulmonary cancer. Biomarkers are either molecular or clinical, and are present in body fluids in either blood or saliva. 

Overall, the protein biomarkers perform better for lung cancer diagnosis due to their involvement in cellular processes. A commonly used set of biomarkers from the literature with sensitivity and specificity is depicted in the Table 4 below. These results are at a specific point of sensitivity and specificity. However, these often fail to provide satisfactory results and are not related to specific types of cancer. Our system recommends these clinical biomarker tests when the classification of benign and malignant nodules is not clearly evident. 

## 5. Experimental Results and Discussion

Extensive experiments were performed to train the models and to validate the performance of the designed architectures. Ten-fold cross-validation was performed for the detection of lung nodules on the LUNA16 [29] dataset, and the LIDC-IDRI annotated data was used for the classification of nodules [30] and application of the LUNA patient level split principle. The designed system was evaluated on both patient and nodule level diagnoses. The proposed models were implemented on NVIDIA TESLA K80 GPU with graphics memory of 12 GB in PyTorch.

### 5.1. Datasets

Publically available datasets LUNA16 and LIDC-IDRI were used for the training of nodule detection and classification, respectively, in the proposed lung cancer detection system. LUNA16 is a subset of LIDC-IDRI dataset which only contains detection annotations confirmed by three or four radiologists. The LIDC-IDRI dataset contains all of the related information on nodules like size, location, results of diagnosis, and other related data to diagnosis from experienced doctors on low-dose CT images in XML files. 3D CT scans are collections of 2D greyscale regular slices in the right order, specified slope, and intercept. The LUNA16 dataset contains only CT scans with uniform slices and well-organized images, while the LIDC-IDRI dataset contains all types of images. The LUNA16 dataset consists of 888 low-dose lung CTs, which contains a total of 754,976 canddiates that are labeled ‘0’ as non-nodule and ‘1’ as nodule. Among these, 36,378 are annotated as nodules by the radiologists, and LIDC-IDRI comprises 1,018 low-dose thoracic CT scans. 

The ground truth nodules for classification were obtained from the annotated LIDC-IDRI dataset, doctors’ mappings, and LUNA16’s annotated nodules. Only those ground truth nodules were considered which have a sufficient score after multiple doctors’ annotations. 

### 5.2. Preprocessing

Thoracic CT scans are huge raw images that need to be meaningful for preprocessing in order to find and classify lung nodules. Efficient preprocessing techniques are necessary to locate the areas of interest, which are extremely small zones in the early stage. On the other hand, the CT images cannot be fed directly to the system for analysis due to huge memory requirements. The most popular publically available LIDC-IDRI dataset contains 1018 DICOM images of approximately 124 GB size, which need a lot of preprocessing and training resources—even a few days—depending upon the system. Lung parenchyma is segmented from the CT scans based on different radio densities of various substances in the image, which are measured in the form of Hounsfield unit (HU).

### 5.3. Training

The nodule detection model was trained through the LUNA16 dataset which contains 888 patient-labeled data, divided into training (710 patients) and validation (178 patients) datasets with augmented data. The nodule classification model was trained on LIDC-IDRI that contains 1018 CT scans in which four radiologists identified 2562 nodules/lesions. 

To address the issues of class imbalance among the FP candidates and true nodules, different augmentation techniques such as translation, rotation, flipping, and cropping were applied to increase the training data and reduce class imbalance. These datasets contain well-labeled data, which is necessary for the traning of the new models. The models were trained by using SGD with mini batch size and 150 epochs. Three different learning rates were used for the whole training process, initially set to 0.01 for 100 epochs, 0.001 for next 30 epochs, and finally 0.0001 for the last 20 epochs with weight decay of 0.0001. To improve the training process and performance, we used ResNeXt with full pre-activation, which led to lower training error with appropriate random weight initialization. 

The LIDC-IDRI dataset was divided into ten sets on the basis of the data split principle for 10-fold cross-validation. The data was separted for training, validation, and testing purposes to train the model efficiently. The weights were learned with SGD. 

The purpose of training is to minimize the difference between the network’s output and ground truth data. There are two main reasons to stop the training process. Firstly, the network has reached its smallest error rate, and secondly, the loss function on validation data does not change anymore.

### 5.4. Nodule Detection

The nodule detector of the designed system was trained on the LUNA16 dataset with 10-fold cross-validation and patient level split. The official evaluation matrix free response receiver operating characteristic (FROC) was used for the LUNA16 dataset to validate the proposed method. 

**Figure 9:**
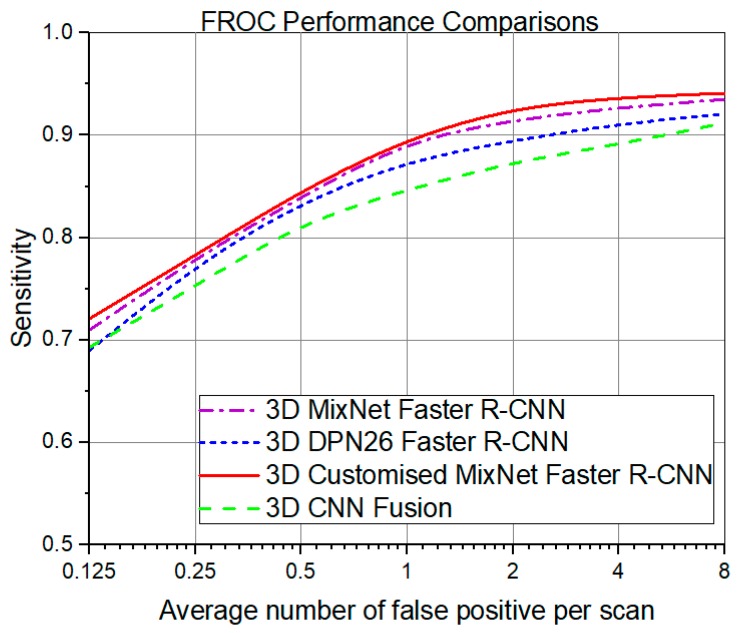
Comparison of different FROC curves obtained from deep learning models.

FROC depicts the sensitivity versus false positive in one scan of the proposed system in Figure 9. The testing phase was performed with detection probability of threshold as -2 prior to applying the sigmoid function. Afterwards, non-maximum suppression (NMS) with intersection over union (IoU) was performed for threshold operation. In Figure 9, the red line represents the proposed 3D CMixNet with faster R-CNN for lung nodule detection, which achieved a FROC score of 94.21% which is higher than the 3D MixNet (94%), with the magenta line showing 3D DPN26 and Faster R-CNN represented with blue line which attained a FROC mark of 92% detection of nodules. The proposed system achieved these results with a lesser number of parameters as compared to 3D DPN26.

### 5.5. Nodule Classification

The nodule classification performance of the designed system was evaluated on LIDC-IDRI on the basis of LUNA16’s dataset split principle of 10-fold cross-validation of patient level data. For classification training, 3250 nodules were used which contain equal numbers of positive and negative nodules. The detected cropped nodules of sizes 32 × 32 × 32 were padded into 36 × 36 × 36. The zero padded nodules were randomly cropped for data augmentation. Due to the minute difference between benign and malignant nodules, 1000 epochs were used with three different learning rates of 0.01, 0.001, and 0.0001. The classification performance for nodules is depicted in Table 5. Results show (in Table 5) that the prosed model achieved more accuracy with less numbers of parameters as compared to the existing ones. 3D CMixNet achieved better feature exploitation by comparing obtained features at each level, and the optimization of features produced better results with GB machine classification.

Automated lung nodule detection and classification techniques have high detection capabilities with large numbers of false positive results; on the other hand, clinical biomarkers have low detection capability and high specificity. When the diagnosis is performed with automated CT analysis techniques in connection with clinical biomarkers, the collective decision helps to reduce false positive results. The detection of nodules in the presence of clinical evidence increases the chances of malignancy, while the detection of nodules without clinical evidence may lead to careful study of the nodule’s morphology before a final decision. The clinical knowledge base also helps to make better decisions with CT analysis techniques.

The performance of the proposed CAD system for lung cancer detection was evaluated based on false positive reduction rate and various statistical measures, such as sensitivity, specificity, accuracy, and area under the receiver operative cure (AUC). AUC values vary from 0.5 to 1. Higher values of AUC indicate significant performance of the system. The statistical measures are represented in the form of mathematical equations to measure system performance. Here, TP, TN, FN, and FP represent true positive, true negative, false negative, and false positive, respectively. Sensitivity measures the TP rate while avoiding FN. Specificity measures the TN rate. Different relations are mentioned below, which are used for the evaluation of lung nodule detection and classification models. The comparison of the results of these parameters are shown in Table 6.

(11) $$\mathrm{Sensitivity}=\frac{TP}{TP+FN}$$

(12) $$\mathrm{Specificity}=\frac{TN}{TN+FP}$$

(13) $$\mathrm{Accuracy}=\frac{TP+TN}{TP+TN+FP+FN}$$

(14) $$\mathrm{Positive}-\mathrm{predctive}-\mathrm{value}=\frac{TP}{TP+FP}$$

(15) $$\mathrm{Negative}-\mathrm{predictive}-\mathrm{value}=\frac{TN}{TN+FN}$$

### 5.6. Computational Complexity

All of the experiments were conducted on NVIDIA TESLA K80 GPU with graphics memory of 12 GB in PyTorch. The computational cost was evaluated in terms of number of parameters and training time of the models in milliseconds per sample. In the detection model, we used eight blocks of CMixNet with bottleneck layers and average pooling to limit the number of parameters and apply faster R-CNN on the learned features for the detection of nodules. In the nodule classification model, thirty blocks of CMixNet were applied, but only on cropped areas with detected nodules which also limited the number of parameters. Training time is related to the architecture of the model, but in life-critical applications, a more important parameter is the error rate rather than training time. Therefore, we selected the best models to reduce the error rates of detection and classification rather than focusing on training time in our designed models—but still, MixNet architecture showed the least number of parameters as compared to other modern CNNs. The running computational cost of the proposed model is minimal, while the training computational cost is almost one week on our GPU, like the other deep learning models. The training computational cost of our detection and classification models are depicted in Table 7.

## 6. Conclusions

The proposed multi-strategy-based lung nodule detection and classification system has the objective of false positive reduction at early stages. The 3D lung CT image was analyzed for the presence of malignant nodules. Firstly, the lung CT image was subjected to 3D Faster R-CNN with CMixNet and a U-Net-like encoder–decoder for the presence of nodules. The detected nodules were further analyzed through 3D CMixNet with GBM to classify the nodule as benign or malignant. Lastly, the deep learning-based nodule classification results were further evaluated with multiple factors, such as patient family history, age, smoking history, clinical biomarkers, size, and location of the detected nodule. A lot of experiments were performed on the publically available LUNA16 and LIDC-IDRI datasets. Results show the superiority of the proposed system with lesser computational cost.

## Figures and Tables

**Table 1:** Relative contribution of risk factors with clinical biomarkers [3].

Factor	Sensitivity (%age)	Specificity (%age)	NPV (%age)
Proteins LG3BP/C163A	97	13	95
Smoking history	97	<5	-
Age	97	8	92
Nodule size	100	13	100
Nodule location	97	<5	-
Nodule speculation	97	<5	-
Physiological symptoms	97	<5	-

**Table 2:** Symptoms appear at different stages in lung cancer.

Physiological Symptoms	Stage 1 (%age)	Stage 2 (%age)	Stage 3 (%age)	Stage 4 (%age)
Body temperature	56–66	32–78	93–97	95–99
Breathlessness	5–54	41–86	89–94	95–100
Pain	26–43	30–63	34–76	43–82
Anxiety	35–47	48–65	66–76	78–95
Irregular heart rate	12–63	19–74	74–96	97–98
Blood pressure	27–42	63–86	87–91	92–94
Fatigue	17–39	28–48	67–78	79–89
Insomnia	37–47	48–62	63–87	88–92
Body weight loss	34–64	44–60	89–93	93–98
Depression	19–31	22–46	37–78	47–83
Constipation	10–20	18–25	26–43	44–60
Anorexia	-	-	36–68	67–78

**Table 3:** Output feature maps at different stages of our CMixNet architecture.

Stage	Output	Weights
Initial input size	96 × 96 × 96, 24	3 × 3 × 3, 24
First CMixNet block	48 × 48 × 48, 48	$\left\{\begin{array}{c} 1\times 1\times 1,24\\ 3\times 3\times 3,24,\\ 1\times 1\times 1,32 \end{array}\begin{array}{c} \left(\mathrm{stride}-2\right)\\ C=32 \end{array}\right\}\times 2$
	
	
Second CMixNet block	24 × 24 × 24, 72	$\left\{\begin{array}{c} 1\times 1\times 1,48\\ 3\times 3\times 3,48,\\ 1\times 1\times 1,56 \end{array}\begin{array}{c} \left(\mathrm{stride}-2\right)\\ C=32 \end{array}\right\}\times 2$
	
	
Third CMixNet block	12 × 12 × 12, 96	$\left\{\begin{array}{c} 1\times 1\times 1,72\\ 3\times 3\times 3,72,\\ 1\times 1\times 1,80 \end{array}\begin{array}{c} \left(\mathrm{stride}-2\right)\\ C=32 \end{array}\right\}\times 2$
	
	
Fourth CMixNet block	6 × 6 × 6, 120	$\left\{\begin{array}{c} 1\times 1\times 1,96\\ 3\times 3\times 3,96,\\ 1\times 1\times 1,104 \end{array}\begin{array}{c} \left(\mathrm{stride}-2\right)\\ C=32 \end{array}\right\}\times 2$
	
	
Upsampling/Deconv. 1	12 × 12 × 12, 216	2 × 2 × 2, 216
Fifth CMixNet block	12 × 12 × 12, 152	$\left\{\begin{array}{c} 1\times 1\times 1,128\\ 3\times 3\times 3,128,\\ 1\times 1\times 1,136 \end{array}\begin{array}{c} \left(\mathrm{stride}-2\right)\\ C=32 \end{array}\right\}\times 2$
	
	
Upsampling/Deconv. 2	24 × 24 × 24, 224	2 × 2 × 2, 152
Sixth CMixNet block	24 × 24 × 24, 248	$\left\{\begin{array}{c} 1\times 1\times 1,224\\ 3\times 3\times 3,224,\\ 1\times 1\times 1,232 \end{array}\begin{array}{c} \left(\mathrm{stride}-2\right)\\ C=32 \end{array}\right\}\times 2$
	
	
Output	24 × 24 × 24, 15	Dropout, p = 0.5 1 × 1 × 1, 64 1 × 1 × 1, 15
	
	

**Table 4:** Important clinical biomarkers with sensitivity and specificity for the presence of cancer.

Biomarkers	Sensitivity (%age)	Specificity (%age)
CYFRA 21-1 (Cytokeratins) [26]	43	89
CEACAM (Carcinoembryonic antigen) [26]	69	68
ProGRP (Pro-gastrin-releasing peptide) [27]	84	95
Carbohydrate Antigen 125 (CA125) [27]	80	40
Carbohydrate Antigen-19.9 (CA-19.9) and Ferritin	78	20
Neuron-Specific Enolase (NSE) [28]	38	40
Squamous Cell Carcinoma Antigen (SCC)	80	67
Proteins LG3BP/C163A [3]	97	13

**Table 5:** Accuracy comparison of nodule classification on public dataset.

Models	Accuracy (%)	Year
Multi-scale CNN [31]	86.84	2015
Nodule level 2D CNN [32]	87.30	2016
Slice level 2D CNN [32]	86.70	2016
Multi-crop CNN [33]	87.14	2017
Vanilla 3D CNN [32]	87.40	2016
Deep 3D DPN [18]	88.74	2017
Deep 3D DPN + GBM [18]	90.44	2017
3D MixNet [24]	88.83	2019
3D MixNet + GBM [24]	90.57	2019
3D CMixNet + GBM	91.13	2019
3D CMixNet + GBM + Biomarkers	94.17	2019

**Table 6:** Performance comparison of TumorNet [34], DFCNet [14], and CMixNet.

Dataset	Method	Sensitivity (%)	Specificity (%)	Accuracy (%)
LIDC-IDRI test data	TumorNet	81.70	85.17	87.41
	DFCNet	80.91	83.22	86.02
	CMixNet	93.97	89.83	88.79
Hospital data	TumorNet	81.49	89.94	81.11
	DFCNet	83.67	96.17	96.33
	CMixNet	98.00	94.35	94.17

**Table 7:** Training computational cost of our detection and classification models.

Mode	Initialization	Optimizer	Regularizer	Learning Rate	Epochs	Training Time
Detection	Random	GSD	BN, Dropout	0.01	100	68 h
Detection	Random	GSD	BN, Dropout	0.001	30	36 h
Detection	Random	GSD	BN, Dropout	0.0001	20	24 h
Classification	Random	GSD	BN, Dropout	0.01	700	52 h
Classification	Random	GSD	BN, Dropout	0.001	200	31 h
Classification	Random	GSD	BN, Dropout	0.0001	100	23 h
